# Detection of Vaccinia Virus in Urban Domestic Cats, Brazil

**DOI:** 10.3201/eid2302.161341

**Published:** 2017-02

**Authors:** Galileu Barbosa Costa, Júlia Bahia Miranda, Gregório Guilherme Almeida, Jaqueline Silva de Oliveira, Mariana Siqueira Pinheiro, Stefanne Aparecida Gonçalves, Jenner Karlisson Pimenta dos Reis, Ricardo Gonçalves, Paulo César Peregrino Ferreira, Cláudio Antônio Bonjardim, Jônatas Santos Abrahão, Erna Geessien Kroon, Giliane de Souza Trindade

**Affiliations:** Universidade Federal de Minas Gerais, Belo Horizonte, Brazil

**Keywords:** vaccinia virus, cowpox, orthopoxvirus, urban, domestic, cats, Brazil, zoonoses, viruses

## Abstract

We investigated possible vaccinia virus (VACV) in urban house cats in Brazil. Serum samples from 6 cats were positive for VACV by PCR, indicating likely VACV circulation among house cats in urban areas of Brazil. This finding highlights the importance of epidemiologic surveillance to avoid outbreaks among urban human populations.

Vaccinia virus (VACV) outbreaks, first reported in Brazil in 1999, affect dairy cattle and humans in rural areas (*1*). Although studies have shown evidence of VACV circulation among several mammal species (*1**–**3*), no consensus exists regarding the role of these animals in the VACV transmission chain or which animal is the natural reservoir. In fact, domestic or wild mammals could be asymptomatic hosts and also contribute to VACV transmission (*3*).

In contrast to VACV, cowpox virus (CPXV) circulates in urban environments in Europe but also in surrounding wild and rural areas (*4*). CPXV is transmitted to humans mainly by cats, which play a link between the natural reservoirs and humans in the urban environment (*4**,**5*). In cats, the clinical course of CPXV infection varies from no symptoms to widespread skin necrotic lesions and can ultimately lead to death (*6*). Some studies have shown serologic evidence of orthopoxvirus infection in cats from Europe and have addressed the role of these animals in orthopoxvirus transmission to humans (*7**,**8*).

Because VACV and CPXV share some epidemiologic features and cats have a prominent role in the urban CPXV transmission chain, we decided to investigate whether urban domestic cats have evidence of exposure to VACV in Brazil. This study was approved by the Animal Experiments Committee of the Universidade Federal de Minas Gerais (registration protocol 315/2014). 

We performed a retrospective study of serum samples from 277 house cats, collected during September 2012–December 2014 in 5 states in Brazil (Technical Appendix Figure 1). The states in this study were those whose veterinary clinics agreed to submit samples. We screened serum samples for neutralizing antibodies by using a >70% plaque-reduction neutralization test (*9*). To detect VACV DNA in serum samples, we performed real-time PCR targeting the C11R and A56R genes (*9*). We directly sequenced A56R fragments in both orientations and in triplicate by using the Mega-BACE sequencer (GE Healthcare, Buckinghamshire, UK). We used ClustalW (http://www.genome.jp/tools/clustalw) and MEGA7 software (http://www.megasoftware.net) to align nucleotide sequences and construct a phylogenetic tree (neighbor-joining method with 1,000 bootstraps).

The cats’ ages ranged from 3 months to 15 years; 150 (54.2%) of the cats were female. Thirteen cats (4.7%) had outdoor access, and 37 (13.4%) were admitted to the veterinary clinic for >1 night. Some cats had clinical illness inconsistent with orthopoxvirus infection, which can overlap with other common dermatologic diseases affecting cats (Technical Appendix Table). Most (8/53 [15.1%]) seropositive cats were from the Pampulha region of the city of Belo Horizonte (Minas Gerais State) (Technical Appendix Figure 1), followed by the eastern region of the city. We detected neutralizing antibodies in 16 animals (5.8%), with titers ranging from 100 to 1,600 neutralizing units/mL; of these, 13 (4.7%) were positive for C11R gene and 6 for A56R gene (Technical Appendix Table). Alignment of the A56R fragments showed high similarity to the homologous gene of VACV isolates from Brazil (Technical Appendix Figure 1). For the phylogenetic tree, we grouped sequences with VACV group 1 and 2 isolates (Figure).

**Figure:**
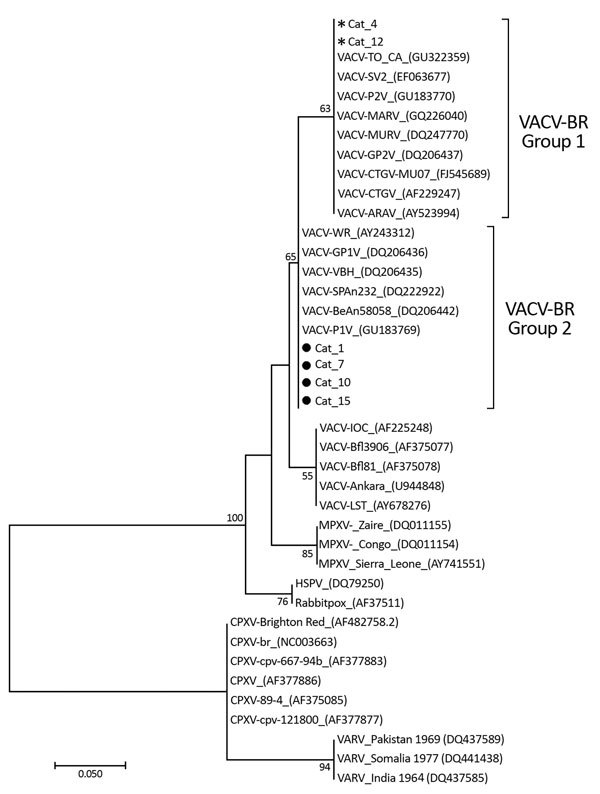
Phylogenetic tree constructed based on nucleotide sequences of orthopoxvirus A56R (hemagglutinin) genes detected in serum samples of 6 house cats house cats with neutralizing antibodies for vaccinia virus, Belo Horizonte, Brazil, September 2012–December 2014. The tree was constructed with A56R gene sequences by using the neighbor-joining method with 1,000 bootstrap replicates and the Tamura 3-parameter model in MEGA7 (http://www.megasoftware.net). Asterisks indicate group 1 vaccinia virus isolates (nonvirulent strains) detected in 2 cats. Black circles indicate group 2 vaccinia virus isolates (virulent strains) detected in 4 cats. Numbers along branches are bootstrap values. GenBank accession numbers are shown for reference isolates. Scale bar indicates nucleotide substitutions per site.

We describe evidence of VACV circulation in cats in an urban environment in Brazil. Many studies have attempted to elucidate VACV outbreaks and risk factors in rural and wild areas (*1**–**3*). Our findings reveal a seropositivity rate of 5.8%, which is lower than the rate observed in a previous study from Norway (*8*) and higher than the rate observed in a study of cats in Austria (*7*). Notably, the Pampulha region, where most seropositive animals were detected, corresponded to areas of relatively low elevation that feature houses with green areas, cottage houses, and ecologic parks, with forested areas making up the remaining portion of the land (Technical Appendix Figure 1). 

Recent data from our research group revealed that capybaras (*Hydrochoerus hydrochaeris*) from the Pampulha region tested positive for VACV (*10*). These data, corroborated by molecular detection of VACV groups 1 and 2 in house cats from Belo Horizonte, further indicate the presence of VACV in an urban environment (Technical Appendix Figure 2). In this study, PCR-positive cats showed no clinical signs that would indicate orthopoxvirus infection at the time of sample collection (Technical Appendix Table), unlike what was observed among cats infected with CPXV in Europe (*4**,**5*). Furthermore, cats 4, 10, and 15 (Technical Appendix Table), in which we detected ongoing VACV DNA, had no clinical signs. Although we detected group 2 VACV (virulent strains) in 4 samples, our findings corroborate the results of Bennett et al. (*6*), which showed that cats infected with VACV had asymptomatic infection. 

Limitations of our study include selection bias of animals; it was not possible to use a convenience sample from the 5 Brazilian states. We were also unable to obtain detailed clinical information of all animals and unable to collect additional clinical samples to better understand the clinical course of VACV infection in cats. In Brazil, no records of VACV-like detection in urban populations are available, despite the fact that VACV was recently found in urban areas (*10*). In fact, potential sources of infection for cat populations (e.g., small rodents) should be considered. Cats could possibly seroconvert without the onset of classical illness. Hence, VACV could be circulating in cats from urban environments. The potential role of cats in infecting humans should be investigated further to determine whether VACV can emerge in urban human populations and pose a threat to public health.

Technical Appendix Diagnostic results for 16 house cats with neutralizing antibodies for vaccinia virus, map of Brazil highlighting studied areas, and nucleotide sequence of the vaccinia virus A56R (hemagglutinin) gene detected in domestic cats compared with homologous sequences of several other orthopoxviruses, Belo Horizonte, Brazil, September 2012–December 2014.
