# Supplementary material for: Detection of Vaccinia Virus in Urban Domestic Cats, Brazil
Source: Emerg Infect Dis. 2017 Feb;23(2):360–2. doi: 10.3201/eid2302.161341 (PMC5324812; doi:10.3201/eid2302.161341)
Supplement: Technical Appendix — Diagnostic results for 16 house cats with neutralizing antibodies for vaccinia virus, map of Brazil highlighting studied areas, and nucleotide sequence of the vaccinia virus A56R (hemagglutinin) gene detected in domestic cats compared with homologous sequences of several other orthopoxviruses, Belo Horizonte, Brazil, September 2012–December 2014. [file 16-1341-Techapp-s1.pdf]

Article DOI: http://dx.doi.org/10.3201/eid2302.161341

Detection of Vaccinia Virus in Urban Domestic Cats, Brazil

Technical Appendix.

Technical Appendix Table. Diagnostic results for 16 house cats with neutralizing antibodies for vaccinia virus, Belo Horizonte, Brazil, September 2012–December 2014*

Cat	$PRNT_{70}$ titer (NU/mL)	qPCR C11R	qPCR A56R†	VACV strain	Clinical signs
1	1:320 (800)	+	+	VACV-BR group 2	Urethral obstruction
2	1:40 (100)	+	–	–	FeLV+
3	1:40 (100)	+	–	–	Hepatic lipidosis
4	1:160 (400)	+	+	VACV-BR group 1	None
5	1:320 (800)	–	–	–	Hepatic lipidosis
6	1:160 (400)	+	–	–	None
7	1:320 (800)	+	+	VACV-BR group 2	Urethral obstruction
8	1:40 (100)	–	–	–	FIV+
9	1:160 (400)	+	–	–	None
10	1:640 (1,600)	+	+	VACV-BR group 2	None
11	1:80 (200)	–	–	–	Sporotrichosis
12	1:80 (200)	+	+	VACV-BR group 1	*Mycoplasma haemofelis*
13	1:40 (100)	+	–	–	None
14	1:640 (1,600)	+	–	–	*Dipylidium caninum*
15	1:640 (1,600)	+	+	VACV-BR group 2	None
16	1:40 (100)	+	–	–	Uncharacterized skin lesions

*BR, Brazil; FeLV, feline leukemia virus; FIV, feline immunodeficiency virus; NU, neutralizing units; qPCR, real-time PCR; $PRNT_{70}$, $\geq$70% plaque-reduction neutralization test; VACV, vaccinia virus; +, positive; –, negative.
†Fragment sequenced.

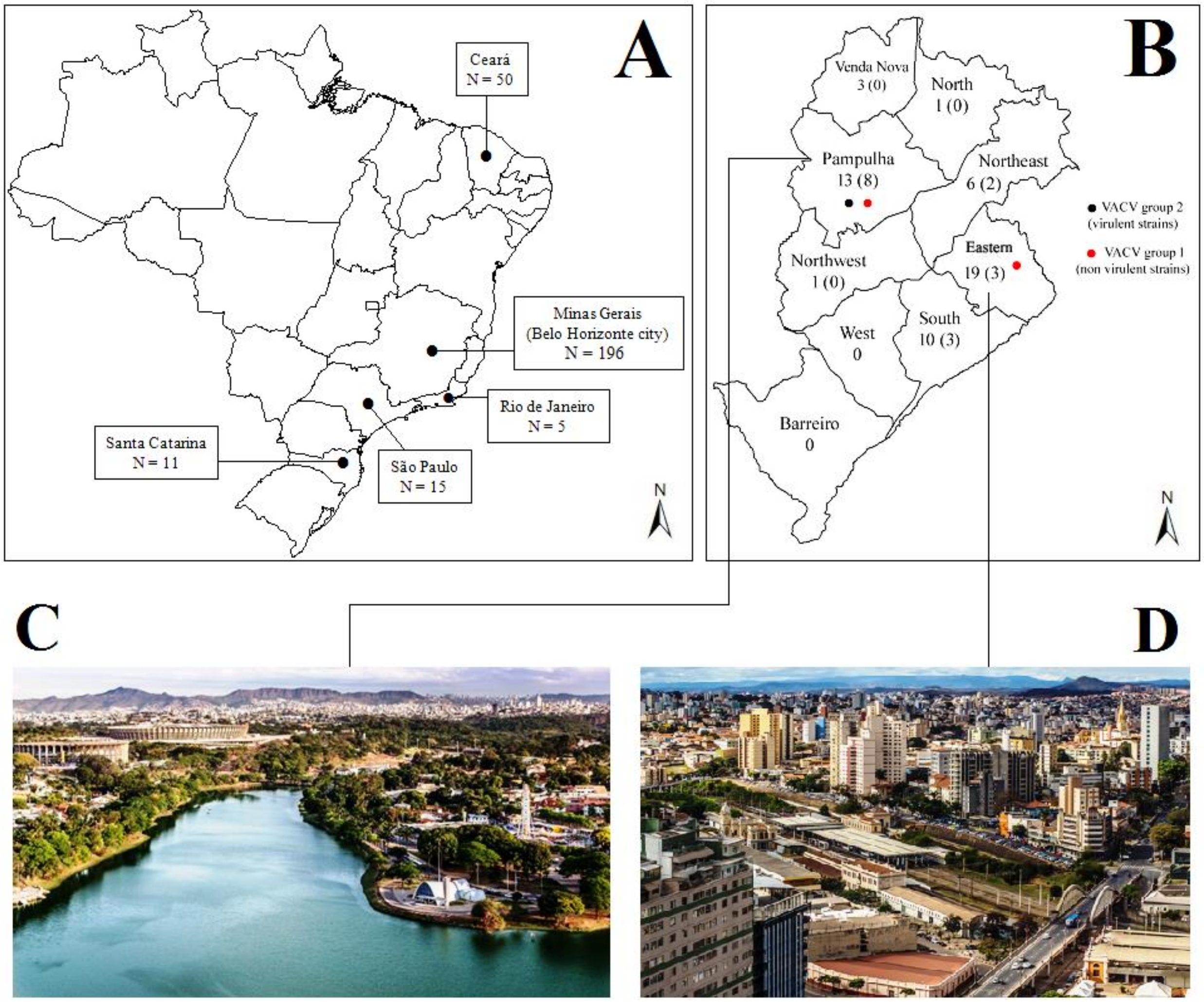

Technical Appendix Figure 1. Map of Brazil highlighting studied areas in black. A) The 5 states in which house cats were sampled. N indicates the number of sampled house cats. B) Map of Belo Horizonte city (located in Minas Gerais State), where most of the house cats were sampled. The map shows the regions in Belo Horizonte including Pampulha and eastern regions. The numbers indicate the number of house cats sampled. Seropositive cats indicated with parentheses. C) Characteristics of Pampulha region. D) Characteristics of eastern regions, respectively.

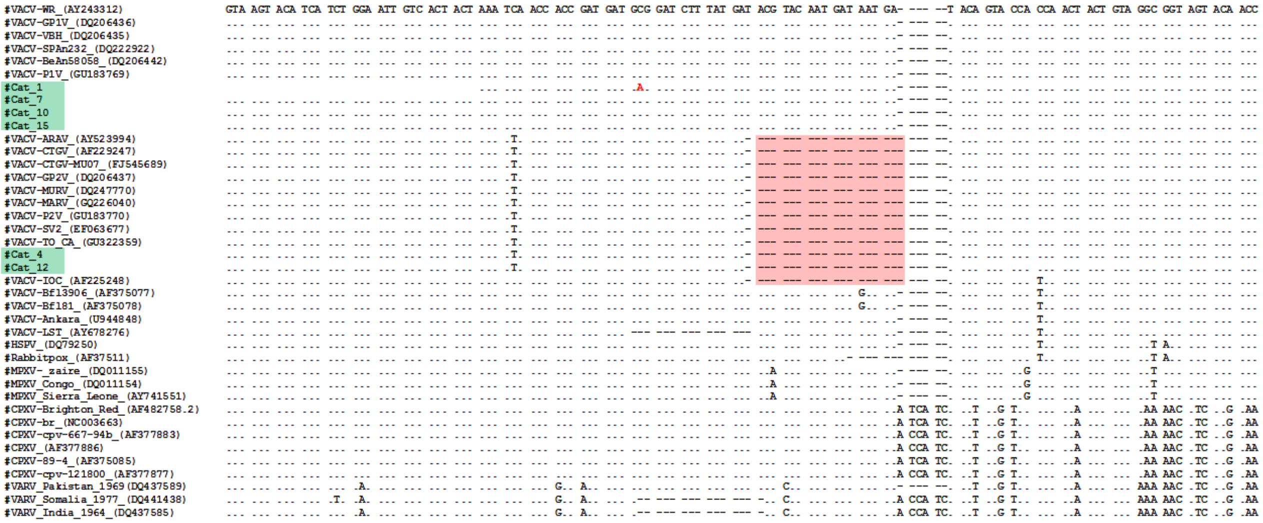

Technical Appendix Figure 2. Nucleotide sequence of the vaccinia virus (VACV)–positive domestic cats A56R (hemagglutinin) genes (green) compared with homologous sequences of several other orthopoxviruses, Belo Horizonte, Brazil, September 2012–December 2014. Strains had the deletion region (red) conserved and were grouped with other VACVs (group 1 and 2) isolated in Brazil. The sample from Cat_1 showed 1 polymorphism (C–A) compared with other VACV isolates.
